# Discriminative Sparse Filtering for Multi-Source Image Classification

**DOI:** 10.3390/s20205868

**Published:** 2020-10-16

**Authors:** Chao Han, Deyun Zhou, Zhen Yang, Yu Xie, Kai Zhang

**Affiliations:** 1 School of Electronics and Information, Northwestern Polytechnical University, Xi’an 710072, China; hanc@mail.nwpu.edu.cn (C.H.); dyzhou@nwpu.edu.cn (D.Z.); zhangkainwpu@mail.nwpu.edu.cn (K.Z.); 2 School of Electronic Engineering, Xidian University, Xi’an 710071, China; sxlljcxy@gmail.com

**Keywords:** domain adaptation, sparse filtering, alternating discriminant optimization, maximum mean discrepancy

## Abstract

Distribution mismatch caused by various resolutions, backgrounds, etc. can be easily found in multi-sensor systems. Domain adaptation attempts to reduce such domain discrepancy by means of different measurements, e.g., maximum mean discrepancy (MMD). Despite their success, such methods often fail to guarantee the separability of learned representation. To tackle this issue, we put forward a novel approach to jointly learn both domain-shared and discriminative representations. Specifically, we model the feature discrimination explicitly for two domains. Alternating discriminant optimization is proposed to obtain discriminative features with an l2 constraint in labeled source domain and sparse filtering is introduced to capture the intrinsic structures exists in the unlabeled target domain. Finally, they are integrated in a unified framework along with MMD to align domains. Extensive experiments compared with state-of-the-art methods verify the effectiveness of our method on cross-domain tasks.

## 1. Introduction

A basic assumption of many machine learning algorithms is that the training and testing data share the same distribution. Now, data sources are more diverse due to the lower costs of data acquisition. For visual images, it may cause inconsistent distribution with some small changes such as lighting conditions, acquisitions, or backgrounds. It is expensive to label each source’s data. Domain adaptation aims to train a robust classifier based on a labeled source domain to predict on an unlabeled target domain [1], which achieved significant progress in image classification [2,3], speech recognition [4,5], person re-identification [6], and many other areas.

An intuitive idea for domain adaptation is to re-weight the training samples and reduce the distance between the source and target domains at the instance level [7]. Another popular way is to reduce the discrepancy between domains at the feature level, which attempts to learn domain-shared representations. Ben et al. pointed out that the transferable features can be obtained by minimizing the distance of domains and maximizing the source margin simultaneously [8]. Based on this theory, many feature-driven domain adaptation methods have been proposed. Pan et al. mapped the data from both domains to high-dimensional Hilbert space and then minimized the domain discrepancy [9]. The measurement employed in [9] is maximum mean discrepancy (MMD) [10], which is capable of characterizing the distance between two sets of samples. Long et al. adapted the marginal probability and conditional probability of the two domains simultaneously by assigning pseudo-labels to the target domain, and achieved more accurate results in an iterative manner [11]. Gong et al. integrated an infinite number of subspaces and characterized changes in consideration of geometric and statistical properties, then proposed a subspace disagreement measure (SDM) to determine the optimal subspace dimension [12]. The combination of distribution matching measures and deep neural networks also achieves remarkable performance. Yosinski et al. studied the transferability of layers in deep neural networks and pointed out that for a particular deep neural network, the first few layers learn general features such as lines or points, and the latter layers learn more specific features [13]. Inspired by this, a series of works have been proposed. Ghifary et al. proposed a domain adaptive neural network for domain adaptation. Compared with a classical neural network, it modeled domain discrepancy explicitly [14]. Tzeng et al. added an MMD adaptation layer to the classic Alexnet [15] for distribution alignment [16]. Long et al. obtained better results by adapting more layers of network and using multi-kernel MMD (MK-MMD) [17]. Inspired by the success of residual structure in image classification, Long et al. proposed residual transfer networks (RTN) to learn cross-layer transferable features [18]. Another interesting idea is adversarial training, which establishes a domain classifier to judge whether a sample comes from the source or target domain. Suppose that a well-trained classifier cannot distinguish samples in two domains; we can say that there are little differences between domains. It differs from other methods in that adversarial training does not measure domain discrepancy with hand-crafted indicators but uses a dynamic classifier. Ganin et al. put forward a gradient reversal layer (GRL) to learn domain-invariant features [19]. Long et al. combined MMD and adversarial training to form a more powerful joint maximum mean discrepancy [20]. Pei et al. considered the multi-mode structures of data and used multiple adversarial networks (each for a class) to align domains [21]. Zhang et al. simultaneously learned both domain-informative and domain-uninformative features through domain collaborative and domain adversarial learning [22].

Many studies focus on dimensionality reduction to facilitate calculation and visualization [23]. Similarly, domain adaptation can also be considered as a feature extraction problem to extract discriminative and shared features. For features with low dimension, it is generally considered that the good features may hold more information, which has been proven to be effective in auto-encoder [24] and reconstruction independent component analysis [25]. The reconstruction error guarantees the completeness of features. However, for the problem of domain adaptation, feature completeness is not necessary because of the inconsistent distribution between the two domains. The knowledge or features are not completely shared between the two domains [1]. Another criterion for extracting features is sparsity, which is generally used as a regular item [26]. Sparse filtering proposed in [27] avoids explicitly modeling raw data, and obtains ideal features by constraining the sparsity of features. Sparse representation-based methods have also made dramatic progress in visual recognition [28].

Most existing methods attempt to shorten the distance between two domains in different feature spaces while maintaining certain statistical characteristics (e.g., variance [9]). Despite the great success achieved, they do not model feature distinctiveness explicitly. In order to handle this problem, we propose a novel dimensionality reduction method for unsupervised domain adaptation in this paper. Apart from reducing the distance between domains, we employ different measurements for source and target domain to obtain discriminative features. Our contributions can be summarized as follows.

- We propose a novel unsupervised domain adaptation solution to reduce domain discrepancy and extract discriminative features simultaneously. Compared to existing works, the proposed method models feature distinctiveness with explicitly constraint. Comparisons with state-of-the-art methods show that our method works well in accuracy and efficiency.
- Alternating discriminant optimization is proposed to obtain discriminative features in the labeled source domain, which utilizes an l2 objective to measure feature distinctiveness. We use a toy example to demonstrate how it works.
- We combine sparse filtering and maximum mean discrepancy into an integrated framework, and propose an unified optimization method with full-batch and mini-batch gradient descent.

The rest of the paper is organized as follows. Section 2 details the domain adaptation problem and related works, then introduces sparse filtering and maximum mean discrepancy. Our method is introduced in Section 3 and experimental evaluation is presented in Section 4. At last, we summarize this paper and discuss future work in Section 5.

## 2. Related Works

In this section, we give a definition of transfer leaning and explain its relationship with domain adaptation. According to whether the labeled samples in the target domain are available, the problem can be divided into semi-supervised and unsupervised domain adaptation. In this paper, we focus on unsupervised domain adaptation, which means that the target domain does not have any labeled samples. Following that, we introduce sparse filtering and maximum mean discrepancy.

### 2.1. Transfer Learning and Domain Adaptation

There are two important concepts in transfer learning, domain and task. A domain, D={X,P(X)}, can be thought of as a set of data, which has a feature space *X* and a marginal probability distribution P(X). The task also has two components, T={Y,f(·)}, *Y* is the label space and f(·) is the mapping function. Traditional machine learning methods have the same domains and tasks between training and testing. When domains or tasks are different, we call it transfer learning (TL). According to the similarity of domain and task, TL can be divided into inductive TL and transductive TL. In this paper, we focus on transductive TL, where the domains are different but related and the tasks are the same.

Domain adaptation can be seen as a kind of transductive TL. Given source data and label (**$X_{s}$,$Y_{s}$**) and target data (**$X_{t}$**), where data in two domains have different distributions, domain adaptation (DA) aims to find the label of target data (**$Y_{t}$**). When the test set is completely unlabeled, it is called unsupervised domain adaptation, which is also the focus of this paper. The mathematical form is defined as follows [11].

(1) $$\left\{\begin{array}{c} P\left(X_{s}\right)\neq P\left(X_{t}\right)\\ P\left(Y_{s}|X_{s}\right)\neq P\left(Y_{t}|X_{t}\right) \end{array}\right.$$

In this paper, we focus on unsupervised domain adaptation which means that target domain has no labels at all. Existing methods try to align features by means of varieties of transformations (e.g., kernel [29], deep neural networks [17]). One crucial thing is how to measure the discrepancy between domains. There are two widely used methods: (a) alignment with moments, whether the first-order moment (maximum mean discrepancy [10]) or the second-order (CORAL [30]); and (b) adversarial training. The main idea is to establish a feature extractor and a domain discriminator simultaneously and train them as generative adversarial nets [31].

### 2.2. Sparse Filtering

Sparse filtering is an effective and simple unsupervised feature extraction method proposed in [27]. It only requires one input parameter: the number of features. Unlike other feature extraction methods, it does not attempt to model the raw data. Instead, it starts with what are good features and directly constrains the extracted features. As a major contribution, the authors gave three principles of the so-called good features.

- Population sparsity: Each example should be represented by only a few active features. Specifically, non-zero elements represent the activation of features, so each sample has few non-zero elements.
- Lifetime sparsity: Good features should be distinguishable. Therefore, a feature is only allowed to be activated in few samples. For example, if we want to classify cats and dogs, the feature of having a tail is activated for all samples, then it is not a good feature.
- High dispersal: It requires each feature to have similar statistical properties across all samples. No one feature should have significantly more activity than the others. This avoids the extraction of features that are only activated on very few samples, and prevents the extraction of similar features.

Furthermore, the authors pointed out that we can obtain ideal representations by jointly optimizing population sparsity and high dispersal, so there is no need to optimize lifetime sparsity explicitly; interested readers can refer to the original paper for more details. Suppose now we have *n* samples, each with *m*-dimensional features that can be written as $x=x_{1},x_{2}\ldots ,x_{n}\in R^{m}$. The optimization of sparse filtering is as follows:

(1) Linear feature extraction. Let $f_{j}^{\left(i\right)}$ represent the *j*th feature of the *i*th sample. $f_{j}^{\left(i\right)}=x^{\left(i\right)}w_{j}$. Then we can use some activation functions to make it more expressive, such as the soft absolute function.

(2) $$f_{j}^{\left(i\right)}=\sqrt{\epsilon +{\left(x^{\left(i\right)}w_{j}\right)}^{2}}\approx \left|x^{\left(i\right)}w_{j}\right|$$

(2) Solving high dispersal. Each feature is divided by the $l_{2}$-norm of the feature on all samples.

(3) $${\tilde{f}}_{j}=\frac{f_{j}}{{∥f_{j}∥}_{2}}$$

Remember that the requirement of high dispersal is that the statistical properties of each feature are similar. This step forces the sum of the squares of all features to be 1 roughly.

(3) Solving population sparsity. Each sample is divided by its own $l_{2}$-norm. Then, we can get the objective function.

(4) $$minimize\sum_{i=1}^{n}{∥\frac{{\tilde{f}}^{\left(i\right)}}{{∥{\tilde{f}}^{\left(i\right)}∥}_{2}}∥}_{1}$$

An advantage of using l2 normalization is to introduce the competition mechanism—that is to say, while some components become larger, some components will become smaller. The result of competition is that the representation becomes sparse.

### 2.3. Maximum Mean Discrepancy

Maximum mean discrepancy (MMD) is widely used to measure the difference between distributions [10]. For domain adaptation problems, researchers pointed out that marginal distribution adaptation can be achieved by minimizing MMD which computes the distance between sample means in the k−dimensional embeddings [9,11].

(5) $$||\frac{1}{n_{s}}\sum_{i=1}^{n_{s}}x_{i}A-\frac{1}{n_{t}}\sum_{j=n_{s}+1}^{n_{s}+n_{t}}x_{j}A|{|}^{2}=tr\left(A^{T}X^{T}M_{0}XA\right)$$

where $M_{0}$ is the MMD matrix that can be computed as:

(6) $${\left(M_{0}\right)}_{ij}=\left\{\begin{array}{ccc} \; & \frac{1}{n_{s}n_{s}},\;\; & x_{i},x_{j}\in D_{s}\\ \; & \frac{1}{n_{t}n_{t}},\;\; & x_{i},x_{j}\in D_{t}\\ \; & \frac{-1}{n_{s}n_{t}},\;\; & otherwise \end{array}\right.$$

Intuitively, the source and target data are integrated together as $X\in R^{(n_{s}+n_{t})\times m}$ where *m* denotes the feature dimension of original data and $n_{s}/n_{t}$ denotes the number of source/target samples. The first $n_{s}$ columns are instances from the source domain and followed $n_{t}$ columns from the target domain. $A\in R^{m\times k}$ is the adaptation matrix which maps the original $x_{i}$ and $x_{j}$ to k−dimensional. As shown on the left of Equation (5), MMD computes the mean vectors for the source and target domains first, then takes the $l_{2}-$norm of the difference between the two vectors.

## 3. Methodology

In this section, we describe the proposed method in detail. First the framework of our approach is introduced. Then, it is followed by a detailed description of the proposed alternating discriminant optimization (ADO) and how the MMD is used in our method. Finally, we summarize the specific optimization problem.

### 3.1. Framework of Discriminative Sparse Filtering

In this paper, we try to learn both discriminative and domain-shared features. Our model consists of two parts: feature transformation and loss function construction. Using the notations defined in Table 1, feature transformation can be described as:

Step 1. Linear feature extraction. Let $f_{act}$ denote the selected activation function. We use the soft absolute function as $f_{act}$ in this paper

(7) $$\begin{array}{cc} \hat{X_{s}} & =f_{act}\left(X_{s}W\right)=\left|X_{s}W+\epsilon \right|\\ \hat{X_{t}} & =f_{act}\left(X_{t}W\right)=\left|X_{t}W+\epsilon \right| \end{array}$$

where ϵ denotes a small number, such as 1*e*-5.

Step 2. Solving high dispersal. Observing the form of $f_{1}$, each row represents a sample, and each column represents a feature. So this step is actually doing a $l_{2}$ column normalization. It is worth noting that we do within-domain normalization instead of cross-domain (which means that using all the samples from two domains to normalize). The idea is to force each feature has similar statistical properties in two domains by setting their $l_{2}$ norm to 1 rudely. As a consequence, a given feature should (a) have similar statistical properties in different domains and (b) be distinguishable over samples in the same domain.

(8) $$\begin{array}{cc} \hat{X_{s}} & =\hat{X_{s}}\circ M_{cs},{\left(M_{cs}\right)}_{ij}=\frac{1}{\sqrt{\sum_{i=1}^{n_{s}}{\left(\hat{X_{s}}\right)}_{ij}^{2}}}\\ \hat{X_{t}} & =\hat{X_{t}}\circ M_{ct},{\left(M_{ct}\right)}_{ij}=\frac{1}{\sqrt{\sum_{i=1}^{n_{t}}{\left(\hat{X_{t}}\right)}_{ij}^{2}}} \end{array}$$

Here, the symbol ∘ represents Hadamard product.

Step 3. Solving population sparsity. Just like the previous step, this step does an $l_{2}$ row normalization.

(9) $$\begin{array}{cc} \hat{X} & =\hat{X}\circ M_{r}\\ where,{\left(M_{r}\right)}_{ij} & =\frac{1}{\sqrt{\sum_{j=1}^{k}{\left(\hat{X}\right)}_{ij}^{2}}},\hat{X}=\left[\begin{array}{c} \hat{X_{s}}\\ \hat{X_{t}} \end{array}\right] \end{array}$$

**Figure 1:**
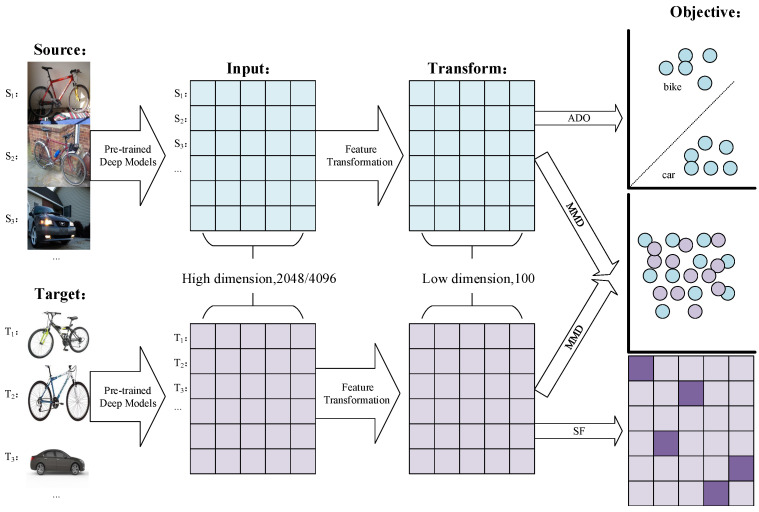
Graphical illustration of the proposed framework.

Notice that steps 2 and 3 do not change the dimension of samples; we can regard them as a specific activation. Based on the descriptions, the transformation from the initial data *X* to $\hat{X}$ is summarized as $\hat{X}=f\left(X\right)$. The loss function can be described as:

(10) $$\begin{array}{cc} L= & \alpha L_{target}\left(f\left(X_{t}\right)\right)+\beta L_{source}\left(f\left(X_{s}\right),Y_{s}\right)\\ \; & +L_{mmd}\left(f\left(X_{s}\right),f\left(X_{t}\right)\right) \end{array}$$

where $L_{target}$ represents the sparse loss on the target domain, $L_{source}$ represents the discriminative loss of the source domain, and $L_{mmd}$ denotes the MMD loss between the source and target features. α,β are the parameters that balance the three objectives. Obviously, $L_{source}$ and $L_{mmd}$ correspond to the two goals presented in [8]. Furthermore, we require the target domain features to be discriminant. A graphical illustration of the framework is shown in Figure 1. Given raw pictures from source and target domains, we first extract their vectorized features with pre-trained deep models, e.g., Alexnet and Resnet. It is worth noting that we do not employ any fine-tuning. Then, they are further reduced in dimension by a linear transformation matrix *W*, after steps 1–3. The objective constructing on the learned representation can be divided into three parts: (1) source domain—objects from different category should far away from each other; (2) target domain—the learned representation should be sparse; and (3) cross-domain—there should not be clear gap among two domains’ samples.

### 3.2. Target Domain Sparsity: Sparse Filtering

In order to obtain discriminative features, we first need an indicator to evaluate the impact of current features on the classification. According to the theory in [32], classification error is the most effective evaluation criterion for feature selection. The specific process is to establish a classifier using the existing features and labels, and then take the classifier error as the discriminant index of the current features. However, there are not any labels in the target domain for unsupervised domain adaptation, which brings difficulties to extract discriminative structures. Since sparse filtering has made remarkable achievements in many areas, in this paper, we introduce sparse filtering for the target domain.

(11) $$L_{target}\left(f\left(X_{t}\right)\right)=\sum_{i=1}^{n_{t}}\sum_{j=1}^{k}{\left(f\left(X_{t}\right)\right)}_{ij}$$

### 3.3. Source Domain Discriminability: Alternating Discriminant Optimization

For the labeled source domain, we can establish a classifier using the transformed features $f(X_{s})$ and labels $Y_{s}$ directly. Different from heuristic feature selection, we hope to solve the optimal transformation matrix by combining the sparsity of the target domain data, which requires that this indicator can be optimized using gradient information. So the classification model whose parameters are solved in an iterative manner (e.g., neural network and SVM) is no longer applicable. In this paper, we use mean square error, then obtain discriminative features by alternately optimizing two parameters.

Suppose that we have source features $F_{s}=f\left(X_{s}\right)\in R^{n_{s}\times k}$ and labels $Y_{s}$. To measure the discriminability of the features, we need $W_{lr}\in R^{k\times 1}$ to map features to label space; here, we use linear mapping function because it can be easily solved without multiple iterations. The objective can be described in mathematical form as:

(12) $$\begin{array}{c} \underset{W,W_{lr}}{minimize}\;\;L=\frac{1}{2}||F_{s}W_{lr}-Y_{s}|{|}_{2} \end{array}$$

A linear regressor maps original data to label space by means of a transformation matrix ($W_{lr}$). Obviously, we actually perform linear regression in the feature space. As mentioned earlier, classification error is the most effective index for feature selection, but it is an $l_{0}$ constraint and makes trouble for optimizing with gradient information. Here, we relax the constraint to $l_{2}$ which is equivalent to linear regression. In general, we hope to measure the discriminability of features by $l_{2}$ constraint and optimize it with gradient descent.

For this two-variable (*W* and $W_{lr}$) optimization problem, it is hard to optimize two parameters simultaneously. So we borrow the ideas of alternating direction method of multipliers (ADMM) [33]. *W* transforms original data to features space where we construct a linear regressor by means of $W_{lr}$. At each iteration, we first solve the linear regressor by normal equation, then update *W* by the chain rule and gradient descent. The specific process is showed in Algorithm 1.
**Algorithm 1:** Alternating Discriminant Optimization.
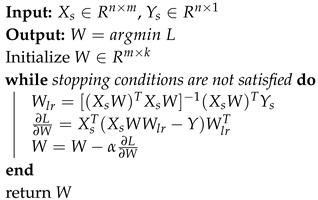


**Figure 2:**
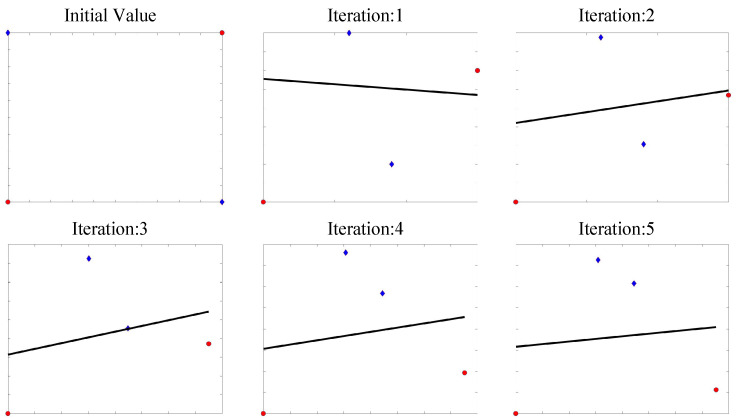
A toy experiment of using ADO for XOR problems.

The main idea of ADO is to find the optimal classifier parameters $W_{lr}$ for each generation of input features, and then optimize the mapping function *W* based on it. With the iterations, the mapping features will have a smaller regression error with the optimal $W_{lr}$. In Figure 2. we show how ADO solves XOR problem. Specifically, we set four examples, i.e., class zero (denoted by blue diamonds): [0,1], [1,0] and class one (denoted by red circular): [0,0], [1,1], which cannot be divided by a single line. ADO computes the optimal decision boundary, then learns a nonlinear mapping to minimize classification error. As the figure shows, samples are mapped into another two-dimensional feature space where they are linearly separable.

Correspondingly, we can formulate $L_{source}$ as follows.

(13) $$L_{source}\left(f\left(X_{s}\right),Y_{s}\right)=\frac{1}{2}||f\left(X_{s}\right)W_{lr}-Y_{s}|{|}_{2}$$

### 3.4. Domain Discrepancy: MMD

We have described how MMD works with linear transformation, but there is a small change in our method. In the previous presentation, we mapped the data to the feature space by multiplying matrix ($x\to A^{T}x$). The case is more complicated here (x→f(x)), but the idea is similar.

(14) $$\begin{array}{cc} L_{mmd} & =||\frac{1}{n_{s}}\sum_{i=1}^{n_{s}}f\left(x_{i}\right)-\frac{1}{n_{t}}\sum_{j=n_{s}+1}^{n_{s}+n_{t}}f\left(x_{j}\right)|{|}^{2}\\ \; & =tr(f^{T}\left(X\right)M_{0}f\left(X\right)) \end{array}$$

where $X=\left[\begin{array}{c} X_{s}\\ X_{t} \end{array}\right]$ denotes the merged data sets.

### 3.5. Optimization

In this section, we give the detailed process to solve three objectives.

#### 3.5.1. Optimization of $L_{target}$

It is the same as applying sparse filtering on the target domain data.

(15) $$\begin{array}{c} \frac{\partial L_{target}}{\partial W}=X_{t}^{T}\left(M_{r}\circ M_{ct}\circ \frac{\partial f_{act}\left(X_{t}\right)}{\partial X_{t}W}\right) \end{array}$$

At each iteration, update $M_{r},M_{ct}$ and then use the updated parameters to calculate the gradient. Notice that we do not give the specific derivation of the select activation functions (soft absolute function); a more general form of the problem is given here, and more activation functions can be used, such as *sigmoid* and *tanh*.

#### 3.5.2. Optimization of $L_{source}$

Based on the derivation in the previous section and the chain rule, we have:

(16) $$\frac{\partial L_{source}}{\partial W}=X_{s}^{T}\left[M_{r}\circ M_{c}\circ \frac{\partial f_{act}\left(X_{s}\right)}{\partial X_{s}}\circ \left(f\left(X_{s}\right)W_{lr}-Y_{s}\right)W_{lr}^{T}\right]$$

where $W_{lr}={\left[f{\left(X_{s}\right)}^{T}f\left(X_{s}\right)\right]}^{-1}f{\left(X_{s}\right)}^{T}Y$ represents the analytic solution of linear regression applied on source features.

#### 3.5.3. Optimization of $L_{mmd}$

We give the derivation of $\frac{\partial L_{mmd}}{\partial f(X)}$; the rest is the same as $\frac{\partial L_{target}}{\partial W}$.

(17) $$\frac{\partial L_{mmd}}{\partial W}=\frac{\partial L_{mmd}}{\partial f(X)}\cdot \frac{\partial f(X)}{\partial W}=f^{T}\left(X\right)M\cdot \frac{\partial f(X)}{\partial W}$$

where *X* consists of $X_{s}$ and $X_{t}$, so Equation (17) provides the gradients of $f(X_{s})$ and $f(X_{t})$. It is worth noting that $M_{r},M_{c}$ are different for the two domains, so we should compute the gradients separately.

**Figure 3:**
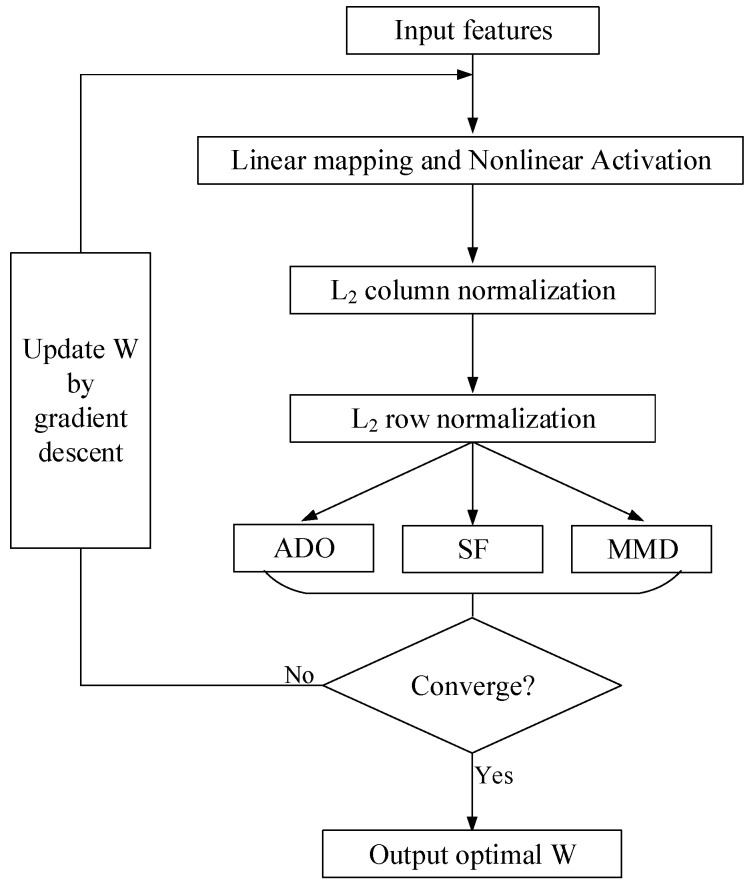
Flowchart of the proposed method.

Given these, we can update *W* with $W=W-\alpha \frac{\partial L_{target}}{\partial W}-\beta \frac{\partial L_{source}}{\partial W}-\frac{\partial L_{mmd}}{\partial W}$, and the flowchart can be found in Figure 3.

## 4. Experiments

In this section, we introduce two data sets for domain adaptation and the experimental settings, then give the results. In addition, we provide an empirical analysis to show the robustness of the proposed method.

### 4.1. Data Set

#### 4.1.1. Office-Caltech10

**Figure 4:**
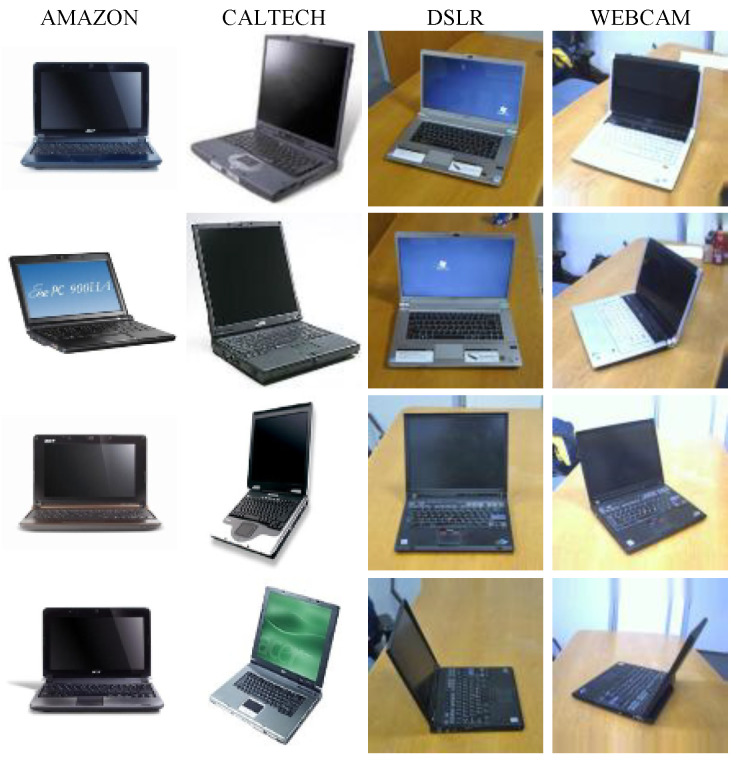
Laptops in different domains.

Office-Caltech10 data set is proposed in [12], which consists of four domains—AMAZON (A), CALTECH (C), DSLR (D), and WEBCAM (W). It comes from the e-commerce website (AMAZON), data set caltech-256 (CALTECH), high-resolution digital camera photo (DSLR), and low-resolution photo (WEBCAM). Each domain has 10 types of objects, including laptop, monitor, and so on. Figure 4. shows the laptop in different domains. It can be seen that there are differences between domains, which brings difficulties to image recognition.

#### 4.1.2. ImageCLEF

ImageCLEF is an online competition for domain adaptation, which has three domains (Caltech (C), Imagenet (I), and PASCAL (P)) and twelve classes of objects.

### 4.2. Experimental Setting

The existing methods can be roughly divided into shallow methods and deep methods. Though our method does not have deep architectures, we choose some deep methods to illustrate its effectiveness. Following [11], we convert the data to 100 dimensions by our method, then use a 1-nearest neighbor for classification. For our method, we set α=0.1 and β=1e−5.

The selected state-of-the-art methods are:

- **Nearest neighbor (NN):** NN is served as a baseline model to check whether the learned representations really work for DA problems.
- **Joint distribution alignment (JDA): [11].** JDA [ICCV2013] adopts pseudo labels to align the conditional distributions of two domains.
- **Correlation alignment (CORAL): [30].** CORAL [AAAI2016] obtains transferable representations by aligning the second-order statistics of distributions.
- **Confidence-aware pseudo-label selection (CAPLS): [34].**CAPLS [IJCNN2019] uses a selective pseudo labeling procedure to obtain more reliable labels.
- **Modified A-distance sparse filtering (MASF): [35].**MASF [Pattern Recognit.2020] employs an L2 constraint combining sparse filtering to learn both domain-shared and discriminative representations.
- **Selective pseudo-labeling (SPL): [36].**SPL [AAAI2020] is also a selective pseudo labeling strategy based on structured prediction.
- **Generalized softmax (GSMAX): [37].** GSMAX [Inf. Sci.2020] aims to learn smooth representation with both labeled source domain and unlabeled target domain.

Follow the experimental setting of JDA and MASF, we set the subspace dimension k=100. For JDA, we set the regularization coefficient λ=1 and the number of iterations T=10. For CAPLS, we set the number of iteration T=10. For MASF, we set the regularization coefficient α=1e−3. For SPL, we set the number of iterations T=11. For GSMAX, we set the regularization factor to 1e−5. It is worth emphasizing that the input features are extracted by deep networks without fine-tuning and no pre-processing strategy is applied in the experiments.

Following the setting of [35,36], we report the classification accuracy on target data as the evaluation metric.

(18) $$\begin{array}{cc} Accuracy & =\frac{\sum_{i=1}^{n_{t}}1\left(\hat{y}\left(x_{i}\right)=y\left(x_{i}\right)\right)}{n_{t}}\times 100\%,\;x\in X_{t}\\ 1\left(\mathrm{case}\right) & =\left\{\begin{array}{cc} 1 & ,\;case\;is\;TRUE\\ 0 & ,\;otherwise \end{array}\right. \end{array}$$

where $\hat{y}$ denotes the predicted label and *y* is the true label, so 0≤Accuracy≤100.

### 4.3. Implementation Details

(1) Initialization. We find that setting the initial value near 0 can significantly improve the convergence. In this paper, we set it to N(0,1)×0.001 where N(0,1) denotes Gaussian distribution. We fix the random number seed to 0 (in MATLAB) for the reproducibility of this paper.

(2) Gradient descent. We set the maximum number of iterations to 200 and the step size to 0.1.

(3) Our code will be available at https://github.com/wobuhuiyingyu/DA_DSF.

### 4.4. Results

In this section, we report the accuracy of the proposed method (abbreviated as DSF for discriminative sparse filtering) and other state-of-the-art works; the results are shown on Table 2 and a detailed comparison can be found on Table 3. From experimental results, we have the following observations:

- DSF vs. NN. According to the results, DSF is significantly better than NN. NN cannot handle the domain discrepancy, thus results in unsatisfying performance. On the other hand, it indicates that our method is able to learn transferable representations.
- DSF vs. CORAL, JDA. DSF is superior to CORAL and JDA. These two methods are classical distribution matching methods, but they have limited considerations on the discrimination of learned representations.
- DSF vs. MASF. MASF is another framework based on sparse filtering, which adopts a modified A distance for domain alignment. Compared to our method, it cannot ensure that the learned representation can be classified easily.
- DSF vs. CAPLS, SPL. Objectively speaking, our method DSF has comparable performance when compared state-of-the-art works, only 0.5% decreasing on average accuracy. It reveals that the proposed discriminative features are applicable for domain adaptation problems.

The difference of sample numbers, also referred as class weight bias, is a fundamental problem for measuring distribution differences. Existing measurements, e.g., MMD and CORAL, employ the first/second/higher order moments to quantify distribution differences, which assume that the source and target data share the same class weights; however, such an assumption does not always hold (like Office-Caltech10). However, our method also yields good classification results. The reason is twofold: (1) the class weight biases are not so severe that they will lead to catastrophic accumulation of errors. (2) There are other regularizations, i.e., the proposed ADO and sparse filtering. The ideal features should be both domain-shared and discriminative, so the negative effects can be further suppressed. Another interesting phenomenon is the different results after changing the order of two domains; this can be explained by the information asymmetry. Imagine that two sets A and B, where A∈B, so if we choose B as training set and A for testing, the model would achieve satisfactory performance. If the order is changed, the model would fail since A cannot provide enough discrimination power.

### 4.5. Empirical Analysis

#### 4.5.1. Ablation Study

For better understanding of the proposed method, we conduct an ablation study to analyze how different components contribute to the final performance. Since there are too few samples for some domains of the Office-Caltech10, e.g., 157 images in total for DSLR, we use ImageCLEF for ablation study only. Compared to original sparse filtering, we proposed two strategies, i.e., MMD for distribution matching and ADO for source discrimination. Through the arrangement and combination of two elements, we can construct $2^{2}=4$ experiments. We use **✓**and **✗**to denote the status of two components, e.g., MMD (**✓**) + ADO (**✗**) indicates that current model is MMD regularized sparse filtering. As Table 4 shows, when the two components are all activated, the method achieves the highest average performance. Adding one component can also improve the final prediction.

#### 4.5.2. Parameter Sensitivity Analysis

In this paper, we introduce two parameters, α and β, to balance the three parts of our objective. α is the coefficient of target sparsity; we hope to preserve the invisible structure of target samples by constraining its sparsity. Similarly, β indicates how much we care about source discriminability.

(1) α=0,β=0.

In this situation, we do not care about the discriminability of both domains. All we need is to reduce domain discrepancy by reducing the MMD loss, which is similar to TCA [9].

(2) α↑,β↑.

**Figure 5:**
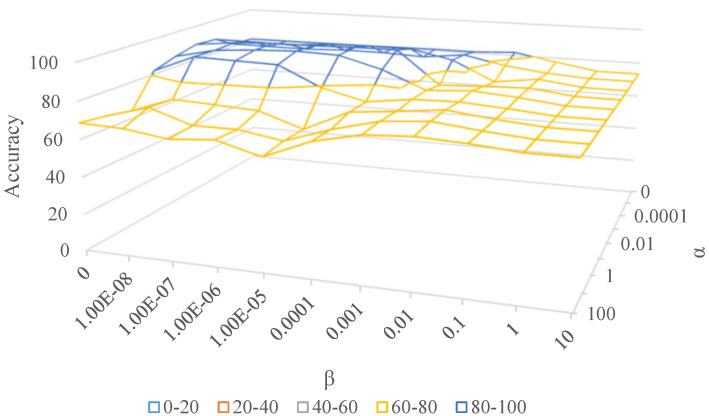
Accuracy (%) with different α and β on Office-Caltech10.

Extended from TCA, we hope to obtain discriminative representations while reducing domain discrepancy in some sense. However, if they are too large, we cannot learn transferable knowledge across domains. As Figure 5 shows, α becomes larger from front to back and β increases from left to right. Obviously, the highest peak occurs in the middle of the surface, which manifests that the proposed two strategies are both necessary. When α/β becomes too large (corresponding to the right and rear of the surface), the accuracy decreases sharply since we pay too much attention to feature discriminability while ignoring the fundamental problem, i.e., distribution matching.

#### 4.5.3. Running Time

Using given notations, the computational cost is detailed as follows: $max(O\left(n_{s}\cdot k^{3}\right),O\left(n_{s}\cdot m\cdot k\right))$ for solving $L_{source}$, $O(n_{t}\cdot m\cdot k)$ for $L_{target}$, and $O({\left(n_{s}+n_{t}\right)}^{2}\cdot k)$ for $L_{mmd}$. In summary, suppose we take *T* as the number of iterations; the overall computation complexity of algorithm is $T\cdot max(O\left(n_{s}\cdot k^{3}\right),O\left(n_{s}\cdot m\cdot k\right),O\left(n_{t}\cdot m\cdot k\right),O\left({\left(n_{s}+n_{t}\right)}^{2}\cdot k\right))$.

In this section, we record the running time (feature extraction + classification with NN) of previous experiments. All algorithms are implemented via MATLAB 2017a and executed on a Windows PC with Intel Core i7 CPU at 3.6GHz and 8GB RAM. Table 5 shows the results. Intuitively, we can see that the proposed method computes faster than most of other works on average running time, especially CAPLS and SPL.

## 5. Discussion

In this section, we discuss the influence of different gradient-based optimization methods on the proposed framework.

### 5.1. Mini-Batch versus Full-Batch

In the previous section, we show how to apply gradient descent for optimizing the proposed method, which means that we need all data ($n_{s}+n_{t}$) for computing. However, real-world applications may have large amount of data so that our computer cannot handle the heavy computation. Consequently, stochastic gradient descent (SGD), which adopts a subset ($k\ll n_{s}+n_{t}$) of data during each iteration, is necessary. In this section, we analyze how mini-batch based optimization may affect our method both theoretically and practically.

#### 5.1.1. Implementation of Mini-Batch-Based Optimization

Mini-batch SGD randomly selects a part of samples to calculate gradients rather than on the whole data set. Similarly, we can solve the proposed framework with mini-batch SGD. Firstly, we should select samples in source and target domain ($MiniX_{s},MiniX_{t}$) separately since MMD needs data from both domains. The batch size can be determined by our computation resource. Then by treating the two mini-batch ($MiniX_{s},MiniX_{t}$) as $X_{s}$ and $X_{t}$, we can update the parameters using gradient descent (showed in Section 3.5. Optimization).

#### 5.1.2. Influence of Mini-Batch SGD

**Figure 6:**
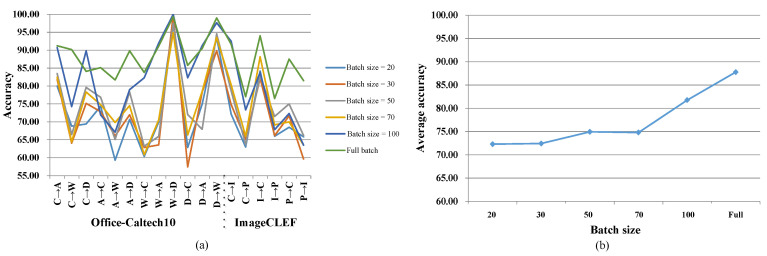
Experiments with different batch sizes. (**a**) Accuracy (%) on every subtask with different batch sizes. (**b**) Average accuracy (%) with different batch sizes.

Here, we analyze how mini-batch SGD affects the proposed method. Since we use random mini-batch instead of the full batch, the sampling error cannot be ignored. For sparse filtering (corresponding to $L_{target}$), as an unsupervised feature extraction method, it requires a diversity of data. In extreme cases, suppose that we have data from the same class. Sparse filtering tries to extract distinguishable features; in other works, it tries to make samples from the same class to be different, which is counterintuitive. For alternating discriminant optimization (corresponding to $L_{source}$), it learns discriminative features with labeled source samples. If the samples belong to the same class in the mini-batch, it outputs meaningless gradients. For MMD (corresponding to $L_{mmd}$), it measures the domain discrepancy with first-order statistics. The sampling error is reflected in the gap between the mean of mini-batch and the full batch ($\tilde{MiniX_{s}}\neq \tilde{X_{s}},\tilde{MiniX_{t}}\neq \tilde{X_{t}}$). To summarize, using mini-batch SGD will lead to performance degradation and the degradation will become larger as the batch size becomes smaller. As Figure 6 shows, the proposed method achieves higher accuracy as the batch size becomes larger, as does the average accuracy. It is worth emphasizing that using mini-batch based optimization is not time-efficient; in fact, it often costs more time. The reason is that we need a small step size and more iterations to train the model, since a min-batch provides a biased estimation of the whole data set. It works when our computer cannot handle the large data set at a time—in other words, it can be seen as a trade-off of time and space.

## 6. Conclusions

In this paper, we propose a novel feature extraction method for unsupervised domain adaptation, which consists of three parts: (a) Since the target domain has no labels, sparse filtering is introduced to capture its discriminative structure in nature. (b) For the labeled source domain, we propose alternating discriminant optimization to directly model the relation of learned representation and labels; a toy experiment of XOR problem shows its validity. (c) We integrate MMD into the framework to reduce domain discrepancy and a unified optimization based on gradient descent is raised. Adequate experiments show that the proposed method is comparable or superior to existing methods. Furthermore, we give a mini-batch based optimization framework such the proposed method can be applied in large-scale problems. In the future, we plan to study how different metrics work to measure domain discrepancy.

## Figures and Tables

**Table 1:** Notations and descriptions used in this paper.

Notations	Description
$D_{s}/D_{t}$	source/target domain
$X_{s}/X_{t}$	original source/target domain data
$Y_{s}/Y_{t}$	source/target domain label
$n_{s}/n_{t}$	number of source/target samples
m/k	original/transformed feature dimension
$\hat{X_{s}}/\hat{X_{t}}$	transformed source/target domain data
f(·)	mapping function, $\hat{X}=f\left(X\right)$
*W*	the transformation matrix to be solved
$W_{lr}$	weight matrix for alternating discriminant optimization
$L_{target}$	objective function for sparsity in the target domain
$L_{source}$	objective function for alternating discriminant optimization in the source domain
$L_{mmd}$	objective function for domain discrepancy
α,β	the balance factors among three objectives

**Table 2:** Performance (accuracy %)on Office-Caltech10 (No.1-12) and ImageCLEF (No.13-18).

No.	Task	NN	JDA	CORAL	CAPLS	MASF	SPL	GSMAX	DSF
1	C→A	85.69	89.77	92.00	90.90	90.81	**92.80**	92.48	91.12
2	C→W	66.10	83.73	80.00	88.83	87.46	85.08	81.02	**91.52**
3	C→D	74.52	86.62	84.70	90.08	89.81	**91.72**	89.81	89.17
4	A→C	70.35	82.28	83.20	80.66	**87.36**	81.39	85.31	83.88
5	A→W	57.29	78.64	74.60	80.69	81.02	**84.07**	81.69	82.03
6	A→D	64.97	80.25	84.10	89.45	86.62	**90.45**	87.26	89.17
7	W→C	60.37	83.53	75.50	**86.62**	85.04	74.00	81.39	81.92
8	W→A	62.53	90.19	81.20	91.38	91.34	**91.96**	77.97	89.35
9	W→D	98.73	**100.00**	**100.00**	**100.00**	99.36	**100.00**	97.45	**100.00**
10	D→C	52.09	85.13	76.80	88.05	85.75	**88.51**	84.95	84.23
11	D→A	62.73	91.44	85.50	92.32	90.40	**93.32**	90.61	91.44
12	D→W	89.15	98.98	99.30	98.66	98.98	**100.00**	98.98	98.30
13	C→I	85.16	92.00	83.00	91.00	89.83	90.83	87.33	**93.16**
14	C→P	69.16	75.50	71.50	77.33	72.83	**78.17**	70.39	75.63
15	I→C	91.16	92.33	88.66	94.17	93.17	94.33	92.83	**95.66**
16	I→P	73.16	77.00	73.66	75.80	76.83	77.50	**78.68**	77.49
17	P→C	81.33	83.83	72.50	90.67	85.33	**91.33**	**91.33**	85.83
18	P→I	74.50	79.16	72.33	85.00	80.83	85.83	**86.67**	82.50
19	AVG	73.28	86.13	82.14	**88.42**	87.38	88.40	86.45	87.91

**Table 3:** An intuitive comparison of average performance (accuracy, %) and average running time (time, s).

	NN	JDA	CORAL	CAPLS	MASF	SPL	GSMAX	DSF
Accuracy	73.28	86.13	82.14	88.42	87.38	88.40	86.45	87.91
Time	0.503	13.114	16.441	1078	7.228	1326	2.499	6.176

**Table 4:** Ablation study. Classification accuracy (%) with different measures.

MMD	ADO	CI	CP	IC	IP	PC	PI	AVG
**✗**	**✗**	91.17	76.48	95.00	75.31	86.83	82.83	84.60
**✗**	**✓**	92.67	76.31	95.00	76.65	87.00	**83.83**	85.24
**✓**	**✗**	92.17	75.63	95.83	76.82	89.00	83.67	85.52
**✓**	**✓**	**93.00**	**78.17**	**96.33**	**77.16**	**89.17**	83.50	**86.22**

**Table 5:** Running time (S) on Office-Caltech10 (No.1–12) and ImageCLEF (No.13–18).

No.	Task	NN	JDA	CORAL	CAPLS	MASF	SPL	GSMAX	DSF
1	C→A	1.134	33.893	25.513	2516.933	13.098	3206.378	3.936	10.515
2	C→W	0.770	16.354	23.638	897.388	9.586	1030.175	2.484	6.755
3	C→D	0.776	13.224	23.356	842.526	8.389	921.195	2.677	5.905
4	A→C	1.240	33.622	23.650	3278.291	12.424	4365.437	3.700	11.097
5	A→W	0.770	12.780	23.045	2870.206	8.399	3528.711	2.412	5.879
6	A→D	0.628	10.275	23.005	2725.151	7.513	3272.032	3.224	4.716
7	W→C	0.655	16.215	22.761	735.878	9.288	833.277	2.229	6.479
8	W→A	0.554	12.702	22.771	1510.084	7.939	1840.393	1.874	5.528
9	W→D	0.239	1.619	22.554	319.998	4.174	369.574	1.612	1.951
10	D→C	0.572	13.368	23.084	519.365	8.080	622.318	1.764	5.375
11	D→A	0.490	10.542	23.049	1336.746	7.332	1751.001	1.731	4.678
12	D→W	0.201	1.631	22.370	241.107	4.257	278.883	1.481	1.982
13	C→I	0.158	10.035	2.991	290.860	4.961	341.601	0.873	6.798
14	C→P	0.155	9.949	2.876	264.467	4.922	304.481	0.835	6.893
15	I→C	0.162	9.989	2.850	293.450	4.953	334.425	0.895	6.666
16	I→P	0.177	9.909	2.801	253.168	4.912	290.072	4.536	6.682
17	P→C	0.179	10.039	2.800	263.583	4.823	302.712	1.760	6.600
18	P→I	0.196	9.897	2.826	249.105	5.051	283.321	6.962	6.674
19	AVG	0.503	13.114	16.441	1078.239	7.228	1326.444	2.499	6.176
